# Supplementary figures and images for: Effects of Microecological Regulators on Rheumatoid Arthritis: A Systematic Review and Meta-Analysis of Randomized, Controlled Trials
Source: Nutrients. 2023 Feb 22;15(5):1102. doi: 10.3390/nu15051102 (PMC10005357; doi:10.3390/nu15051102)

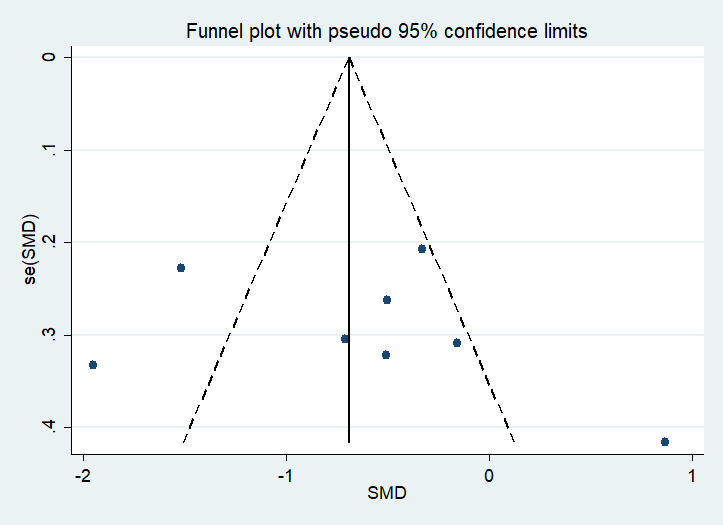

Supplement: Supplementary file 1 [file nutrients-15-01102-s001.zip › Figure S1 Funnel plot of DAS28.tif]

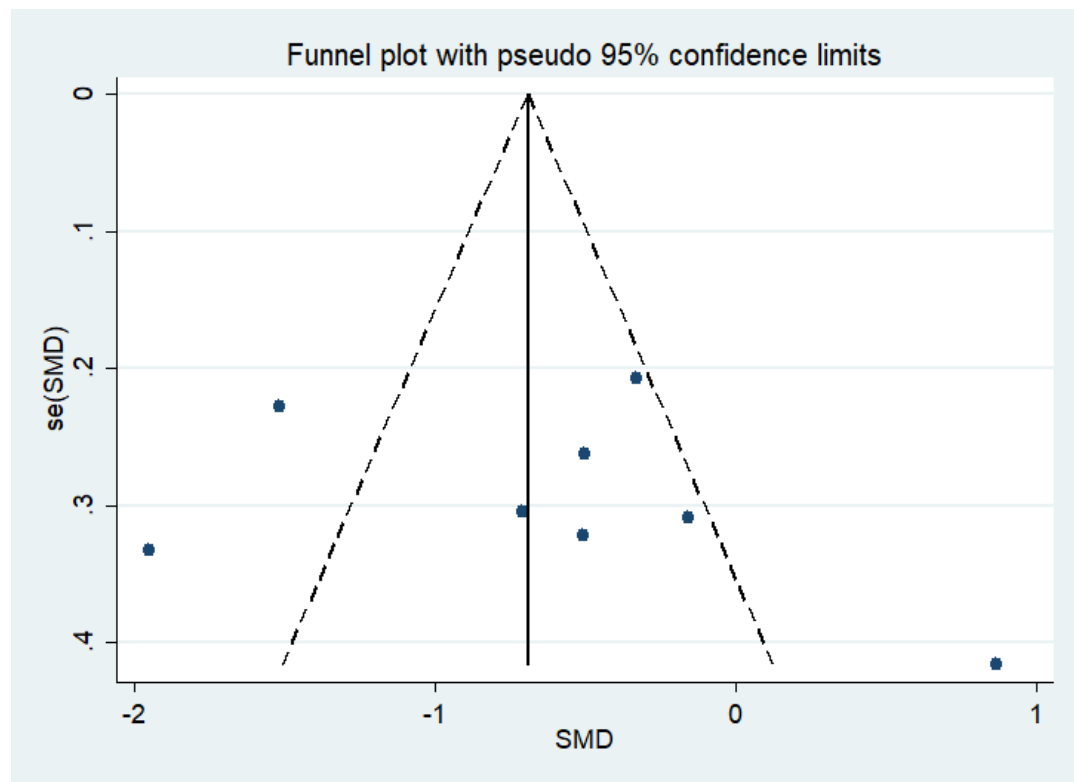

(A)

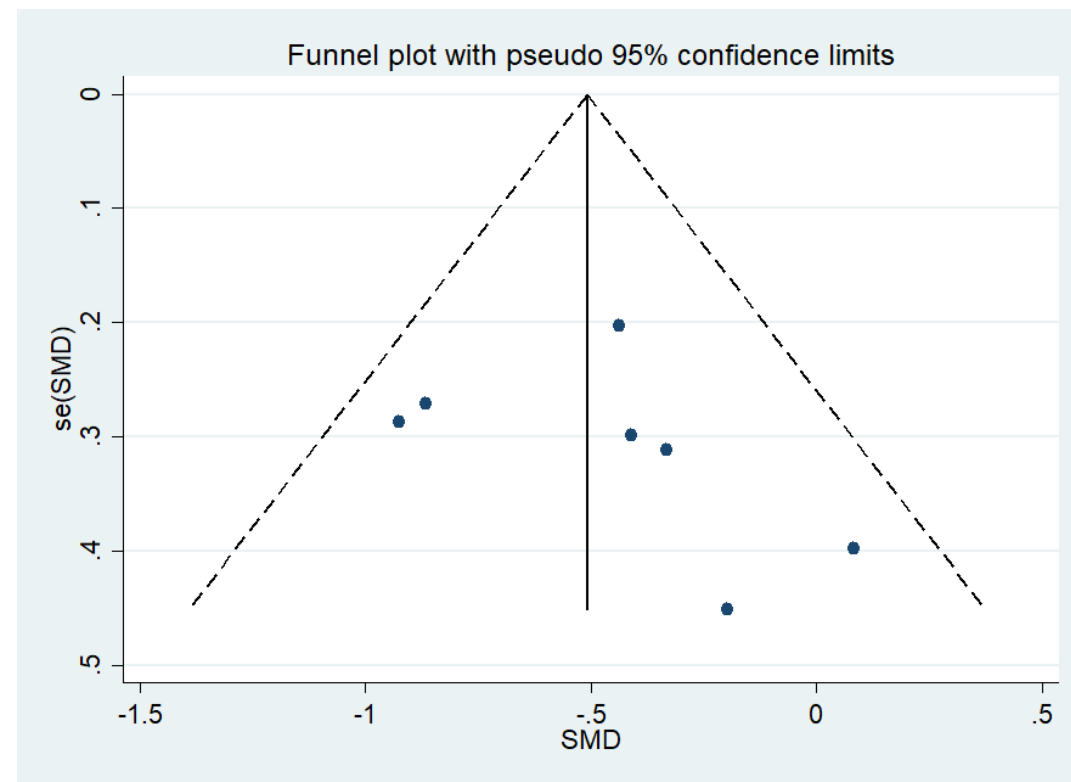

(B)

Figure S1: Funnel plot of DAS28 and CRP. (A) DAS28, (B) CRP

Supplement: Supplementary file 1 [file nutrients-15-01102-s001.zip › Figure S1.pdf]

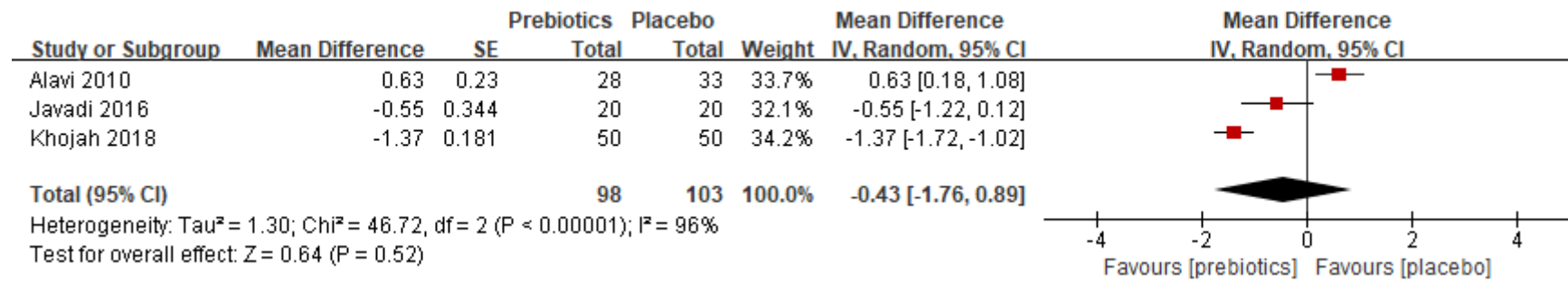

Figure S2: Forest plot of DAS28 variations in subgroup analysis of prebiotics

Supplement: Supplementary file 1 [file nutrients-15-01102-s001.zip › Figure S2.pdf]

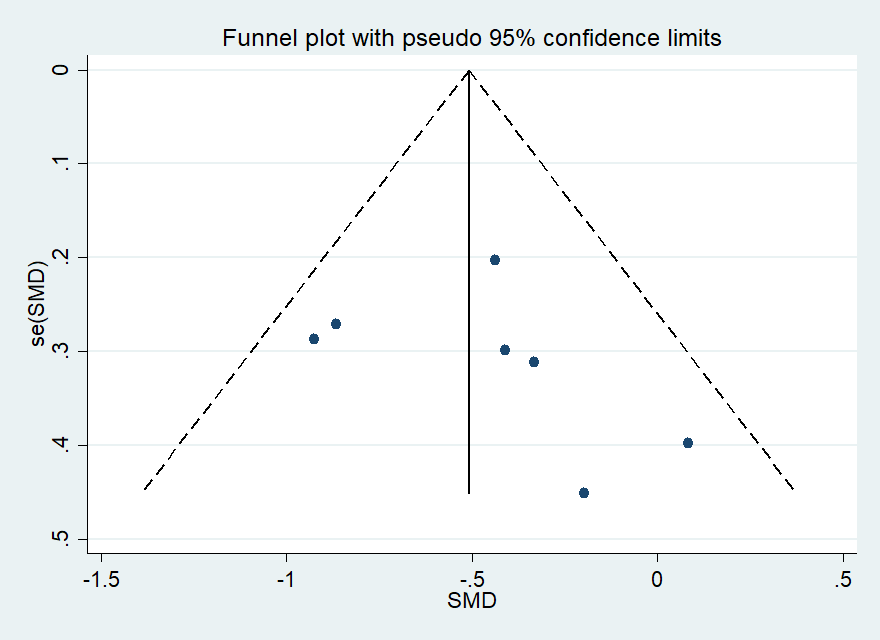

Supplement: Supplementary file 1 [file nutrients-15-01102-s001.zip › Funnel plot of CRP.tif]
